# Plasma Big Endothelin-1 Level Predicted 5-Year Major Adverse Cardiovascular Events in Patients With Coronary Artery Ectasia

**DOI:** 10.3389/fcvm.2021.768431

**Published:** 2021-11-29

**Authors:** Zhongxing Cai, Haoyu Wang, Sheng Yuan, Dong Yin, Weihua Song, Kefei Dou

**Affiliations:** ^1^Cardiometabolic Medicine Center, Fuwai Hospital, National Center for Cardiovascular Diseases, State Key Laboratory of Cardiovascular Disease, Chinese Academy of Medical Sciences and Peking Union Medical College, Beijing, China; ^2^Department of Cardiology, Fuwai Hospital, National Center for Cardiovascular Diseases, State Key Laboratory of Cardiovascular Disease, Chinese Academy of Medical Sciences and Peking Union Medical College, Beijing, China

**Keywords:** coronary artery ectasia, endothelin, cardiovascular death, myocardial Infarction, independent predictor

## Abstract

**Background:** Coronary artery ectasia (CAE) is found in about 1% of coronary angiography and is associated with poor clinical outcomes. The prognostic value of plasma big Endothelin-1 (ET-1) in CAE remains unknown.

**Methods:** Patients with angiographically confirmed CAE from 2009 to 2015, who had big ET-1 data available were included. The primary outcome was 5-year major adverse cardiovascular events (MACE), defined as a component of cardiovascular death and non-fatal myocardial infarction (MI). Patients were divided into high or low big ET-1 groups using a cut-off value of 0.58 pmol/L, according to the receiver operating characteristic curve. Kaplan-Meier method, propensity score method, and Cox regression were used to assess the clinical outcomes in the 2 groups.

**Results:** A total of 992 patients were included, with 260 in the high big ET-1 group and 732 in the low big ET-1 group. At 5-year follow-up, 57 MACEs were observed. Kaplan-Meier analysis and univariable Cox regression showed that patients with high big ET-1 levels were at increased risk of MACE (9.87 vs. 4.50%; HR 2.23, 95% CI 1.32–3.78, *P* = 0.003), cardiovascular death (4.01 vs. 1.69%; HR 2.37, 95% CI 1.02–5.48, *P* = 0.044), and non-fatal MI (6.09 vs. 2.84%; HR 2.17, 95% CI 1.11–4.24, *P* = 0.023). A higher risk of MACE in the high big ET-1 group was consistent in the propensity score matched cohort and propensity score weighted analysis. In multivariable analysis, a high plasma big ET-1 level was still an independent predictor of MACE (HR 1.82, 95% CI 1.02–3.25, *P* = 0.043). A combination of high plasma big ET-1 concentrate and diffuse dilation, when used to predict 5-year MACE risk, yielded a C-statistic of 0.67 (95% CI 0.59–0.74).

**Conclusion:** Among patients with CAE, high plasma big ET-1 level was associated with increased risk of MACE, a finding that could improve risk stratification.

## Introduction

Coronary artery ectasia (CAE) comprises different morphological manifestations of abnormal luminal dilation of the coronary artery (1) and the incidence of CAE ranges from 0.3 to 5% (2). It is defined as abnormal coronary dilation of at least 1.5 times the adjacent normal segment (3). This rare phenomenon is associated with poor clinical outcomes and increased risk of death, non-fatal myocardial infarction (MI), and major adverse cardiovascular events have been observed (4, 5). Previously, our cohort study indicated that diffuse CAE was associated with worse long-term outcomes, compared with focal CAE, which is also known as coronary artery aneurysms (CAA)(6). However, the risk predictors of coronary artery ectasia are not well studied and current studies are limited because of their relatively small sample size.

Big endothelin-1 (ET-1) is the precursor protein of ET-1. It has the same biological importance as ET-1, a higher concentration, and longer half-life in the peripheral blood (7). Previous studies have identified high levels of big ET-1 as a risk factor for poor prognosis in cardiovascular diseases including atrial fibrillation, acute myocardial infarction, and left ventricular non-compaction cardiomyopathy (8–10). It has also been reported as a biomarker for predicting the presence of coronary artery ectasia (11). Whether high plasma big ET-1 levels are a prognostic predictor for patients with CAE remains unknown. In the present study, we aimed to determine the prognostic value of big ET-1 in patients with CAE and to verify if an elevated plasma big ET-1 level is an independent predictor of adverse outcomes in patients with CAE.

## Materials and Methods

### Study Population

Among consecutive patients undergoing coronary angiography in Fuwai Hospital from January 2009 to December 2015, patients with CAE were identified by searching for the terms “coronary artery ectasia” or “aneurysm” in the procedure reports. The angiographic criteria of CAE were defined as: 1) abnormal dilation of more than 1.5 fold the diameter of adjacent normal segments; or 2) if the adjacent normal segment was not available, CAE was defined according to the normal reference value of the corresponding segment from data in age-sex matched patients with normal coronary angiography as previously reported (5) (Supplementary Table 1). The exclusion criteria were: (1) insignificant dilated vessel and the max diameter was < 1.5 times the reference diameter; (2) coronary artery fistula; (3) stent-related coronary artery aneurysms; (4) known autoimmune disease; (5) missing imaging files; (6) valvular heart disease; (7) history of CABG; and 8) plasma big endothelin-1 level not measured. The baseline information of all study objects including demographic features, smoking habits, medical history, family history, medications at discharge, and revascularization strategy were obtained from the hospital's electronic medical records system. This study was approved by the ethics committee of Fuwai Hospital and was conducted in accordance with the Declaration of Helsinki. Informed consent was obtained from all patients.

### Coronary Angiography Evaluation

The angiograms were screened by 2 experienced interventional cardiologists to confirm CAE. CAE was classified as diffuse if the lesion involved more than 1/3 of the vessel length, which was consistent with previous studies (12, 13). Focal CAE or CAA were defined as focal dilation that involved < 1/3 of the artery length. The maximum diameter of the dilated vessel of each patient was measured using quantitative coronary angiography with Qangio XA version 7.3 (Medis, Leiden, Netherlands). Markis classification was used to evaluate anatomical characteristics of CAE (14). Diffuse ectasia of 2 or 3 vessels was classified as type I, diffuse disease in 1 vessel and localized disease in another vessel as type II, diffuse ectasia of 1 vessel only as type III, and localized or segmental ectasia as type IV. Moreover, the SYNTAX scores (15) were calculated for each patient to quantify the severity of combined coronary artery disease.

### Laboratory Measurements

The plasma levels of big ET-1 concentrations in peripheral venous blood were quantified using the Big Endothelin-1 ELISA Kit (NO. BI-20082H; Biomedica, Wien, Austria), following the standard protocol. The intra-assay coefficients of variation values of the Big Endothelin-1 ELISA Kit were, ≤ 5%. The inter-assay coefficients of variation values of the Big Endothelin-1 ELISA Kit were ≤ 4%.

Additionally, the results of plasma high sensitivity C-reactive protein (hsCRP) level, which was reported as a risk predictor in patients with CAE in a previous study (16), were also collected from the hospital's electronic medical records system.

### Outcomes and Follow-Up

Follow-up was conducted annually using standardized questionnaires by telephone interviewers who were blind to the clinical information of the study objects since 2014. The primary outcome was 5-year major adverse cardiovascular events (MACE), which were a component of cardiovascular death and non-fatal MI. The secondary outcome included individual components of the primary outcome, that is, cardiovascular death and non-fatal MI alone.

### Statistical Analysis

Normally distributed continuous data were expressed as mean ± standard deviation and compared using the *t* test. Continuous variables with non-normal distribution were summarized as median (interquartile range, IQR) and compared using the Mann-Whitney test. Categorical variables were expressed as counts (composition ratio), and compared using the Chi-square test or Fisher exact test if appropriate. 1.11% of the plasma hsCRP levels were missing data and handled by single imputation with median. Receiver operating characteristic (ROC) curve analysis was performed and the plasma big ET-1 value with maximum Youden index was selected as the cut-point for 5-year MACE risk stratification. The study population was divided into high and low big ET-1 level groups using this cut-point value. Survival analysis was performed using the Kaplan–Meier method and comparisons between the 2 groups were applied by the log-rank test. Cox proportional hazard regression was conducted to access hazard ratio and multivariable Cox regression was applied to control potential confounding. Moreover, Propensity score (PS) matching, PS weighting, and subgroup analysis served as sensitivity analysis. We used a multivariable logistic regression model to estimate propensity scores, with the big ET-1 level group as the dependent variable and the following variables as covariates: age, gender, body mass index (BMI), hypertension, diabetes, dyslipidemia, peripheral arterial disease, smoking status, acute MI, previous MI, previous PCI, family history of CAD, LVEF, eGFR, combined CAD, location of dilated vessels, diffuse dilation, maximum diameter, the SYNTAX score, medication, and concomitant revascularization. These variables were chosen either because of the statistically significant difference in baseline characteristics between groups or clinical relevance with adverse events. PS Matching was performed using the optimal pair matching with a 1:2 ratio. Propensity score weighting was performed using standardized mortality ratio weighting (SMRW). A standardized difference of < 0.1 indicated a good balance after the PS method. A two-tailed *P* value < 0.05 was regarded as statistical significance. All analyses were performed by R 4.1.0 (R Foundation for Statistical Computing, Vienna, Austria) and SPSS Statistics Version 25 (IBM Corp., Armonk, NY).

## Results

### Baseline and Angiographical Characteristics

**Figure 1:**
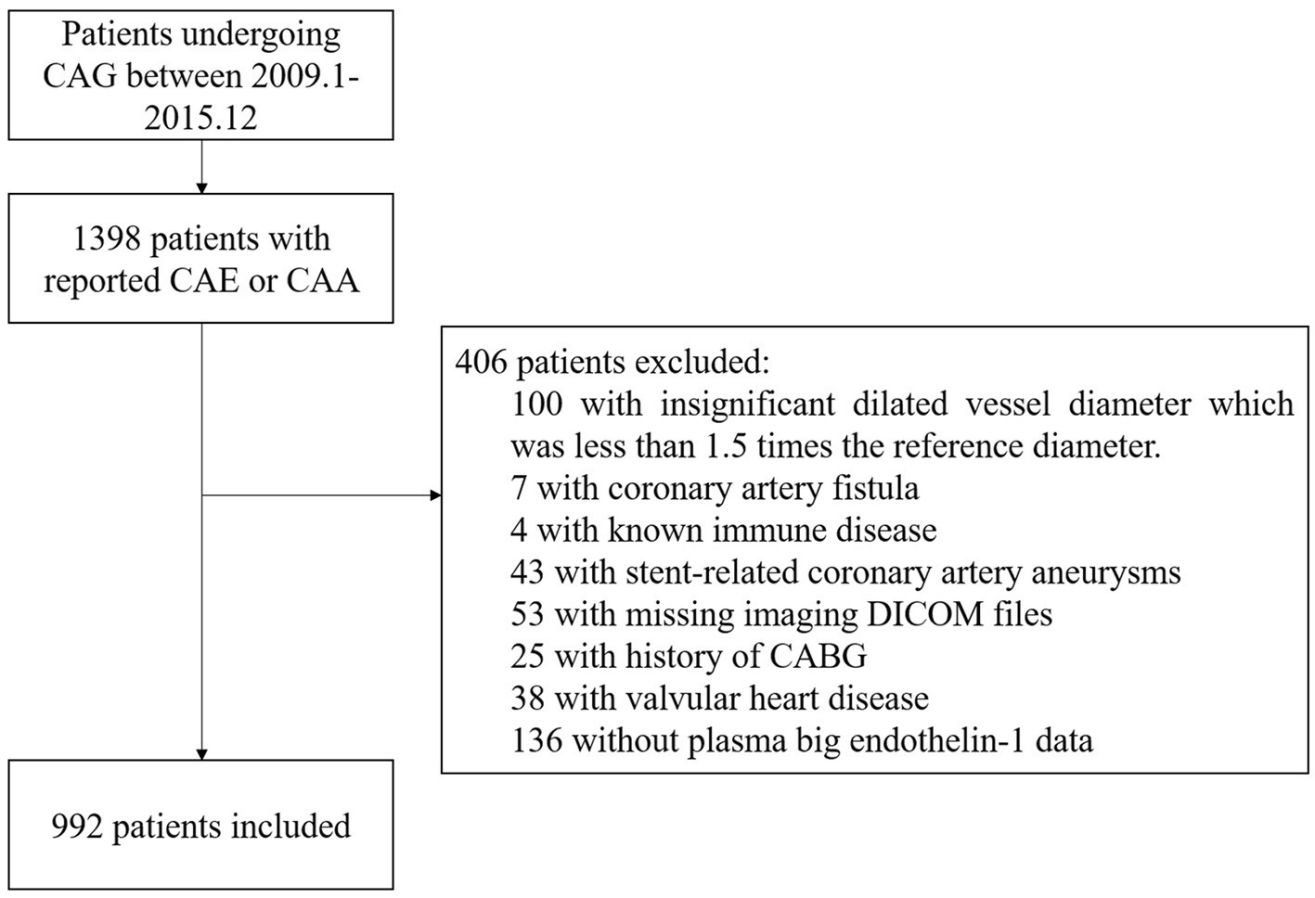
Flowchart of the study subjects. CAA, coronary artery aneurysm; CAE, coronary artery ectasia; CAG, coronary artery angiography.

A total of 922 consecutive patients with CAE were included in this cohort study, indicated by the flowchart shown in Figure 1. From 2009 to 2015, the reported incidence of CAE ranged from 0.83 to 1.36% per year in Fuwai hospital (Supplementary Figure 1). ROC curve analysis indicated 0.58 pmol/L as the cut-point of plasma big ET concentration for 5-year MACE risk stratification (Supplementary Figure 2A). Patients with plasma big ET-1 concentration > 0.58 pmol/L were classified as the high big ET-1 level group and those with a big ET-1 concentration ≤ 0.58 pmol/L entered the low big ET-1 level group. Patients in the high big ET-1 level group tended to have a higher age, less PAD, less current smoking, higher hsCRP, and less statin use at discharge (Table 1). For angiographic characteristics, RCA was the most common dilated vessel, followed by LAD, LCX, and LM in both groups (Table 2). There was no significant difference in angiographic characteristics between the two groups. The maximum diameter of dilated vessels seemed slightly shorter in the high big ET-1 group but did not reach statistical significance (5.38 vs. 5.47 mm, *P* = 0.071).

**Table 1:** Baseline characteristics of the study population.

	**low big ET-1 level group (Big ET-1 ≤ 5.8 pmol/L)**	**high big ET-1 level group (Big ET-1 > 5.8 pmol/L)**	***P*** **-value**
Patient No.	732	260	
Male	611 (83.47)	227 (87.31)	0.171
Age	56.56 ± 10.89	58.75 ± 11.29	0.006
BMI	26.54 [24.27, 29.06]	26.53 [24.22, 29.26]	0.904
Clinical presentation			0.349
Asymptomatic	15 (2.05)	5 (1.92)	
Stable angina	250 (34.15)	79 (30.38)	
Unstable angina	331 (45.22)	119 (45.77)	
NSTEMI	39 (5.33)	12 (4.62)	
STEMI	83 (11.34)	42 (16.15)	
Dyspnea	6 (0.82)	0 (0.00)	
Palpitation	8 (1.09)	3 (1.15)	
Acute MI	122 (16.67)	54 (20.77)	0.164
Previous MI	210 (28.69)	66 (25.38)	0.347
Previous PCI	178 (24.32)	59 (22.69)	0.658
Diabetes	161 (21.99)	63 (24.23)	0.513
Hypertension	476 (65.03)	176 (67.69)	0.483
Dyslipidemia	471 (64.34)	168 (64.62)	0.998
Peripheral arterial disease	57 (7.79)	8 (3.08)	0.013
Family history of CAD	134 (18.31)	39 (15.00)	0.266
Current smoker	279 (38.11)	58 (22.31)	<0.001
LVEF	62.60 [58.00, 67.00]	60.00 [57.00, 65.00]	0.008
LVID	50.00 [47.00, 53.00]	50.00 [46.00, 54.00]	0.446
Big ET-1 concentration	0.25 [0.18, 0.37]	0.77 [0.65, 0.91]	<0.001
hsCRP	1.75 [0.92, 3.41]	2.32 [1.30, 6.16]	<0.001
EGFR	116.06 ± 24.56	110.24 ± 28.91	0.002
Aspirin	713 (97.40)	253 (97.31)	1.000
Clopidogrel	507 (69.26)	167 (64.23)	0.157
Ticagrelor	18 (2.46)	1 (0.38)	0.067
Statins	676 (92.35)	223 (85.77)	0.003
ACEI/ARB	389 (53.14)	155 (59.62)	0.084
β-blocker	628 (85.79)	221 (85.00)	0.834
CCB_DHP	176 (24.04)	69 (26.54)	0.473
CCB_non-DHP	142 (19.40)	52 (20.00)	0.905
Nitrates	612 (83.61)	228 (87.69)	0.141
Concomitant PCI	317 (43.31)	117 (45.00)	0.689
Concomitant CABG	109 (14.89)	38 (14.62)	0.995

*Values are mean ± SD, n (%), or median [interquartile range] unless otherwise stated. ACEI, angiotensin-converting enzyme inhibitors; ARB, Angiotensin Receptor Blockers; BMI, body mass index; CABG, coronary artery bypass grafting; CAD, coronary artery disease; DHP, dihydropyridine; EGFR, estimated glomerular filtration rate; ET, endothelin; LVEF, left ventricular ejection fraction; hsCRP, high sensitivity C-reactive protein; LVID, left ventricular internal dimension; MI, myocardial infarction; NSTEMI, non–ST-segment elevation myocardial infarction; PCI, percutaneous coronary intervention; STEMI, ST-segment elevation myocardial infarction*.

**Table 2:** Angiographic characteristics of the study population.

	**low big ET-1 level group**	**high big ET-1 level group**	***p*** **-value**
Patient No.	732	260	
Combined CAD			0.624
None	83 (11.34)	22 (8.46)	
Single vessel	145 (19.81)	52 (20.00)	
Double vessels	194 (26.50)	72 (27.69)	
Three vessels	251 (34.29)	85 (32.69)	
LM only	2 (0.27)	0 (0.00)	
LM + single vessel	4 (0.55)	3 (1.15)	
LM + double vessels	8 (1.09)	4 (1.54)	
LM + three vessels	45 (6.15)	22 (8.46)	
LM or 3 vessels disease	310 (42.35)	114 (43.85)	0.729
SYNTAX score	14.00 [7.00, 21.00]	14.00 [7.75, 21.50]	0.425
SYNTAX score level			0.653
low (≤ 22)	573 (78.28)	197 (75.77)	
mid (>22 and ≤ 32)	125 (17.08)	51 (19.62)	
high (>32)	34 (4.64)	12 (4.62)	
LM ectasia	97 (13.25)	24 (9.23)	0.112
LAD ectasia	311 (42.49)	109 (41.92)	0.932
LCX ectasia	289 (39.48)	93 (35.77)	0.326
RCA ectasia	438 (59.84)	164 (63.08)	0.398
Markis classification			0.469
Type I	51 (6.97)	12 (4.62)	
Type II	158 (21.58)	60 (23.08)	
Type III	175 (23.91)	69 (26.54)	
Type IV	348 (47.54)	119 (45.77)	
Diffuse dilation	384 (52.46)	142 (54.62)	0.599
Maximum Diameter	5.47 [4.63, 6.30]	5.38 [4.56, 6.12]	0.071

*Values are mean ± SD, n (%), or median [interquartile range] unless otherwise stated. CAD, coronary artery disease; LAD, left anterior descending artery; LCX, Left circumflex artery; LM, left main; RCA, right coronary artery; SYNTAX, Synergy Between PCI With Taxus and Cardiac Surgery*.

### Clinical Outcomes

**Figure 2:**
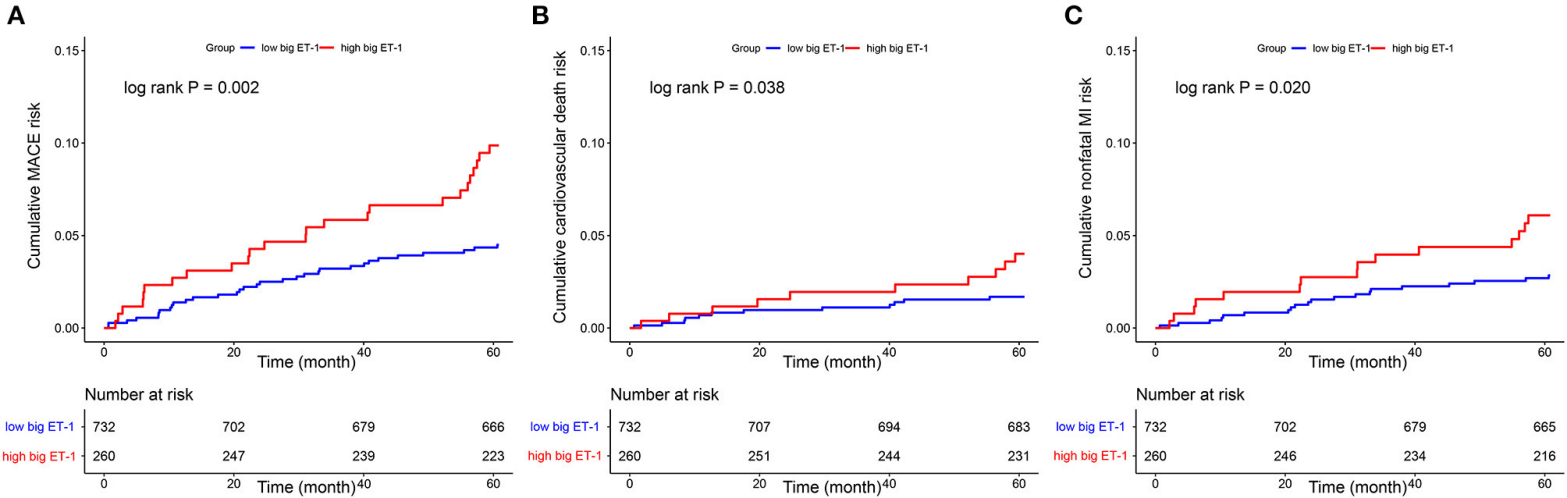
Kaplan–Meier curves showed cumulative event risk of MACE **(A)**, cardiovascular death **(B)**, and nonfatal MI **(C)**. MACE, major adverse cardiovascular event; MI, myocardial infarction; ET, endothelin.

Follow-up data were available for 95.56% (948 of 992) of the eligible patients at 5-years after index angiography. A total of 57 MACE were observed, including 22 cardiovascular death and 35 non-fatal MI. Patients with high big ET-1 levels were associated with significantly higher incidences of MACE (9.87 vs. 4.50%, log rank *P* = 0.002), cardiovascular death (4.01 vs. 1.69%, log rank *P* = 0.038), and non-fatal MI (6.09 vs. 2.84%, log rank *P* = 0.020). Kaplan-Meier curves were used to estimate the event rate of the primary and secondary outcomes, as shown in Figure 2. After adjusting for variables including age, gender, BMI, hypertension, diabetes, dyslipidemia, peripheral arterial disease, current smoker, acute MI, previous PCI, previous MI, family history of CAD, LVEF, eGFR, SYNTAX score, combined CAD, LM ectasia, LAD ectasia, LCX ectasia, RCA ectasia, diffuse dilation, maximum diameter, medications, concomitant revascularization and hsCRP, patients in the high big ET-1 group were still at higher risk of MACE (HR 1.82, 95% CI 1.02–3.25, *P* = 0.043) in multivariable Cox regression. When plasma big ET-1 levels were regarded as a continuous variable, it was still an independent predictor of MACE (HR 2.47, 95% CI 1.10–5.57, *P* = 0.029). The summary of primary outcomes is shown in Table 3, with secondary outcomes in Table 4. Additionally, diffuse dilation, which we found to be a prognostic factor previously (6), was also associated with a higher risk of MACE in both univariable (HR 2.13, 95% CI 1.21–3.75, *P* = 0.009) and multivariable Cox regression (HR 4.12, 95% CI 1.96–8.63, *P* < 0.001) in the current study. The C-statistic estimation of diffuse dilation for predicting 5-year MACE was 0.59 (95% CI 0.53 to 0.66, *p* = 0.009) (Supplementary Figure 2B). Adding plasma big ET-1 to predict 5-year MACE risk yielded a C-statistic of 0.67 (95% CI 0.59–0.74) (Supplementary Figure 2C). Besides, hsCRP, which was found to be a predictor of an adverse event in CAE and other cardiovascular diseases, was also associated with increased MACE in the current study (HR 1.09, 95%CI 1.01–1.16, *P* = 0.014).

**Table 3:** Summary of primary end-point events and hazard ratio of the high big-ET-1 level group.

	**Sample size**		**MACE**		
**Type of analysis**	**high big ET-1 level group**	**low big ET-1 level group**	**Event No**.	HR* (95% CI)	***P*** **-value**
Unadjusted	260	732	25 vs. 32	2.23 (1.32–3.78)	0.003
Matched	260	520	25 vs. 25	2.02 (1.16–3.52)	0.013
Weighted	260	739.50	25 vs. 36.36	1.98 (1.11–3.53)	0.021
Multivariable Model 1^+^	260	732	25 vs. 32	2.15 (1.23–3.76)	0.007
Multivariable Model 2^++^	260	732	25 vs. 32	1.82 (1.02–3.25)	0.043

* *Hazard ratio derived from Cox regression model*.

+ *Multivariable COX regression Model 1 used variables including age, gender, BMI, hypertension, diabetes, dyslipidemia, peripheral arterial disease, current smoker, acute MI, previous PCI, previous MI, family history of CAD, LVEF, eGFR, SYNTAX score, combined CAD, LM ectasia, LAD ectasia, LCX ectasia, RCA ectasia, diffuse dilation, maximum diameter, medications, concomitant revascularization*.

++ *Model2 adjusted for variables in model 1 and hsCRP*.

**Table 4:** Summary of secondary outcomes.

**Type of analysis**	**cardiovascular death**			**non-fatal MI**		
	**Event No**.	HR* (95%CI)	***P*** **-value**	**Event No**.	HR* (95%CI)	***P*** **-value**
Unadjusted	10 vs.12	2.37 (1.02–5.48)	0.044	15 vs.20	2.17 (1.11–4.24)	0.023
Matched	10 vs. 11	1.83 (0.78–4.30)	0.168	15 vs.14	2.19 (1.06–4.54)	0.035
Weighted	10 vs.13.97	2.05 (0.84–4.98)	0.113	15 vs.22.39	1.95 (0.91–4.18)	0.084
Multivariable Model 1^+^	10 vs.12	1.76 (0.67–4.62)	0.250	15 vs.20	2.11 (1.04–4.27)	0.038
Multivariable Model 2^++^	10 vs.12	1.45 (0.52–4.00)	0.478	15 vs.20	1.86 (0.91–3.84)	0.091

* *hazard ratio for high big ET-1 group derived from COX regression model*.

+ *Multivariable COX regression Model 1 used variables including age, gender, BMI, hypertension, diabetes, dyslipidemia, peripheral arterial disease, current smoker, acute MI, previous PCI, previous MI, family history of CAD, LVEF, eGFR, SYNTAX score, combined CAD, LM ectasia, LAD ectasia, LCX ectasia, RCA ectasia, diffuse dilation, maximum diameter, medications, concomitant revascularization*.

++ *Model2 adjusted for variables in model 1 and hsCRP*.

### PS Matching, PS Weighting, and Subgroup Analysis

**Figure 3:**
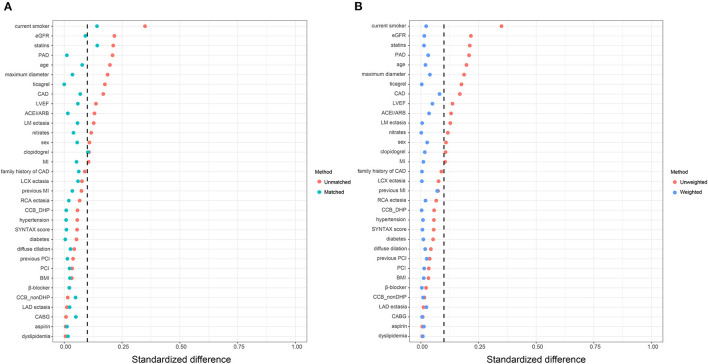
Covariates balance in the study cohort. **(A)** Standardized difference before and after propensity score matching. **(B)** Standardized difference before and after propensity score weighting. ACEI, angiotensin-converting enzyme inhibitors; ARB, Angiotensin Receptor Blockers; BMI, body mass index; CABG = coronary artery bypass grafting; CAD, coronary artery disease; DHP, dihydropyridine; EGFR, estimated glomerular filtration rate; LVEF, left ventricular ejection fraction; MI, myocardial infarction; PCI, percutaneous coronary intervention.

**Figure 4:**
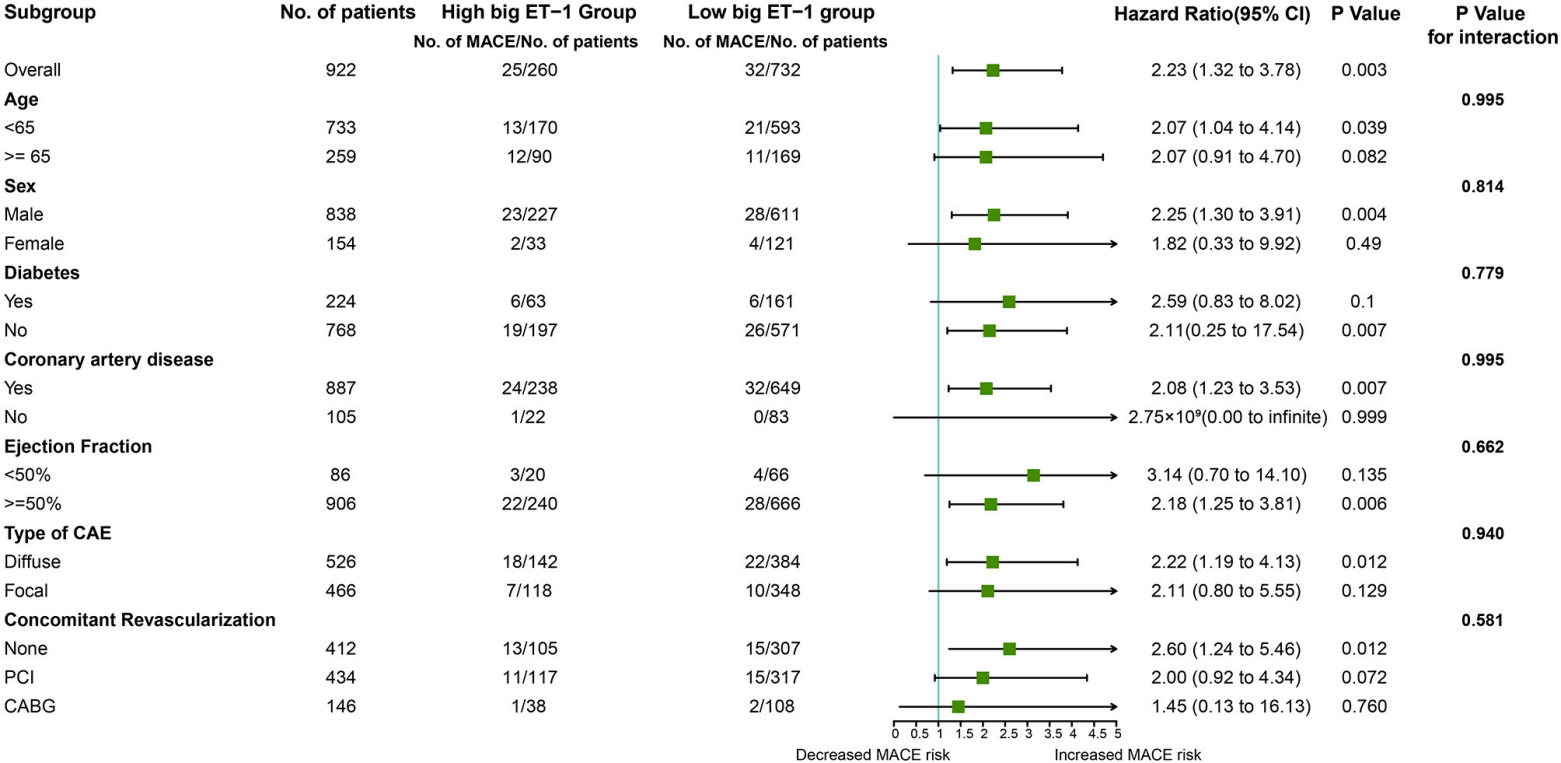
Hazard ratio of MACE among subgroups. ET, endothelin.

Consistently, increased risk of MACE in the high big ET-1 group was observed using the propensity score method. The covariates were well balanced, especially after SMRW where the standardized differences of all the covariates were < 0.1 (Figure 3). As shown in Table 3, matched and weighted data still showed significant higher risks of MACE (HR 2.02, 95% CI 1.16–3.52, *P* = 0.013; HR 1.98, 95% CI 1.11–3.53, *P* = 0.021, respectively) in the high big ET-1 group. The detailed results of the secondary outcomes are shown in Table 4. Subgroup analyses defined by age, sex, diabetes, coronary artery disease, LVEF, type of CAE, and concomitant revascularization between the high big ET-1 group and low big ET-1 group were performed (Figure 4). The trend toward increased risk of MACE in the high big ET-1 group was consistently obtained among all subgroups and no significant interaction effect was found.

## Discussion

This cohort study demonstrated that a high plasma big ET-1 level was associated with an increased risk of MACE in patients with CAE. To the best of our knowledge, this is the first study to evaluate the prognostic value of plasma big ET-1 level in a large cohort of patients with CAE.

CAE was reported to be detected in only 0.3 to 5% of coronary angiography, the number of contemporary studies in this disease was limited and most had a small sample size (4, 5). At the national center for cardiovascular diseases in China, the number of patients undergoing coronary angiography in Fuwai hospital increased from 1,1543 to more than 25,000 per year from 2009 to 2015, which allowed us to study this rare phenomenon in a larger sample compared with previous studies. The incidence of CAE in the angiography report ranged from 0.83 to 1.36% per year in Fuwai hospital. Generally, the characteristics of the current study population were consistent with previous studies. For example, most patients with CAE were male and combined with atherosclerosis. RCA was the most common dilated vessel followed by LAD and LCX (17).

Previously, we found that diffuse dilation predicted poor long-term outcomes in 595 patients with CAE (6). The present study reconfirmed the association between diffuse dilation and adverse cardiovascular events in this larger population. Consistent with another previous study (16), hsCRP was also found to be associated with an increased risk of adverse cardiovascular events in this article. More importantly, we found another predictor, plasma big ET-1 level, was associated with poor clinical outcomes and improved risk stratification in these patients. A big ET-1 level of >0.58 pmol/L was significantly related to an increased risk of major adverse cardiovascular events, suggesting that a high level of plasma big ET-1 could be a potential indicator of a patient's prognosis. Furthermore, increased risk of MACE for the high big ET-1 group was consistently observed in the PS matched cohort, PS weighted data, and subgroup analysis in this study. The higher incidences of non-fatal MI and cardiovascular death were also observed in univariable Cox regression but not in multivariable regression. This might be the result of the limited sample size for this rare disease and the relatively small numbers of each secondary endpoint.

A previous study suggested that plasma big ET-1 level was significantly higher in patients with isolated CAE (11). However, the pathophysiological mechanisms of big ET-1 in CAE were still unknown. Several mechanisms might explain the association among elevated big ET-1 levels, coronary artery ectasia, and unfavorable prognosis.

Big ET-1 is a 39-amino acid precursor of ET-1 and is mainly produced by vascular endothelial cells, vascular smooth muscle cells, and fibroblasts (Supplementary Figure 3). Big ET-1 is converted by the membrane-bound enzyme ET converting enzyme-1 into the functional, mature 21-amino acid form, ET-1. ET-1 binds to two cell surface receptors, ET receptor subtype A (ET_A_R) and subtype B (ET_B_R). ET_A_ receptors are only located on vascular smooth muscle cells and mediate constriction, while ET_B_ receptors are located on both vascular smooth cells, where they mediate vasoconstriction, and vascular endothelial cells, where they contrarily mediate dilation through nitric oxide (NO) release (18). ET-1 is considered to be an important mediator in vessel remodeling, ultimately leading to major changes in cellular and tissue architecture (19). In smooth muscle cells and fibroblasts, it modulates the expression of extracellular matrix and matrix metalloproteinases (MMPs), which may lead to the promotion of tissue remodeling and fibrosis (20). Notably, MMPs are important in the development of coronary aneurysms (21) and aortic aneurysms (22), and transgenic expression of MMP-2 induces coronary artery ectasia in mice models (23). Therefore, it is a reasonable hypothesis that big ET-1 participates in the pathophysiology of CAE. Aneurysmal diseases in other arteries, such as abdominal aortic aneurysms, were closely related to the occurrence of CAE. Endothelin-1 overexpression induced aortic aneurysms in mice by increasing oxidative stress, inflammatory cell infiltration, and matrix metalloproteinase-2 in perivascular fat, vascular wall, and atherosclerotic lesions (24). ET-1 also independently predicts the growth of stable abdominal aortic aneurysms (25). This demonstrated the important role of ET-1 in aneurysmal diseases and suggested the potential mechanistic role of ET-1 in CAE.

Except for the potential etiological role of big ET-1 in CAE, this biomarker has been found to be a poor prognosis factor in many cardiovascular diseases including atrial fibrillation, acute myocardial infarction, and left ventricular non-compaction cardiomyopathy (8–10). Moreover, ET-1 is a key player in endothelial dysfunction and is associated with systemic inflammation (26). Assessment of microcirculation disturbance in patients with coronary ectasia indicated micro thrombotic embolism and microcirculation disturbance (27). ET-1 plays a cardinal role in vascular tone regulation and coronary microvascular dysfunction through two major G-protein-coupled receptors ET_A_R and ET_B_R (18), which might also contribute to the progression of CAE and increased risk of an adverse cardiovascular event. However, the detailed pathogenesis of ET-1 in CAE remains unclear and needs to be explored in the future.

To date, the optimal medical therapy of CAE remains unknown, and high-risk patients might benefit from anticoagulation rather than antiplatelet therapy (2). Case reports also suggested the potential benefits of anticoagulation in some high-risk patients but it is still unclear which patients are at high risk and in need of anticoagulation (28, 29). Symptoms of the patients varied from asymptomatic to acute coronary syndrome and the prognosis varied in different patients. Hence the risk stratification of patients with CAE is warranted and might be useful to guide treatment strategies. In the future, intensive treatment of such high-risk patients might be valuable, including anticoagulation and strict risk factor control, but further comparative study must be performed to assess the effect of intensive therapeutic strategies.

Although the association of elevated big ET-1 with increased MACE risk is demonstrated in our present study, several limitations should be acknowledged. First, the limitation of a single-center observational cohort study must be recognized and the result should be verified in other cohorts in the future. Second, the sample size and event number were relatively small. In multivariable analysis, the association between big ET-1 and cardiovascular death might not be detected due to weakened statistical efficiency. Third, many patients were combined with coronary artery disease, hypertension, or other diseases, which might affect the outcomes. Finally, genetic data was not available in the current study, thus the relation between genetic information, big ET-1 levels, and clinical outcomes in patients with CAE remain unknown.

## Conclusion

Our study demonstrated that the level of big ET-1 was an independent predictor of MACE in patients with CAE. It might be useful to help risk prediction and develop risk stratification protocol for patients with CAE.

## Data Availability Statement

The raw data supporting the conclusions of this article will be made available by the authors, without undue reservation.

## Ethics Statement

The studies involving human participants were reviewed and approved by Ethics Committee of Fuwai Hospital. The patients/participants provided their written informed consent to participate in this study.

## Author Contributions

ZC, WS, and KD: study design, interpretation of results, and revision of the manuscript. DY, WS, and KD: angiography review and patient enrollment. HW and SY: data collection. ZC: data analysis. ZC and KD: preparation of the manuscript. All authors contributed to the article and approved the submitted version.

## Funding

This study was supported by the Chinese Academy of Medical Sciences Innovation Fund for Medical Sciences (2020-I2M-C&T-B-056) and the Chinese National Key Research and Development Project (Grant No. 2018YFC1315600).

## Conflict of Interest

The authors declare that the research was conducted in the absence of any commercial or financial relationships that could be construed as a potential conflict of interest.

## Publisher's Note

All claims expressed in this article are solely those of the authors and do not necessarily represent those of their affiliated organizations, or those of the publisher, the editors and the reviewers. Any product that may be evaluated in this article, or claim that may be made by its manufacturer, is not guaranteed or endorsed by the publisher.
