# Supplementary material for: Plasma Big Endothelin-1 Level Predicted 5-Year Major Adverse Cardiovascular Events in Patients With Coronary Artery Ectasia
Source: Front Cardiovasc Med. 2021 Nov 29;8:768431. doi: 10.3389/fcvm.2021.768431 (PMC8667227; doi:10.3389/fcvm.2021.768431)
Supplement: Supplementary file 1 [file Data_Sheet_1.docx]

Supplementary Material

# Supplementary Figures and Tables

## Supplementary Figures

**Supplementary Figure 1. The numbers of reported CAE in Fuwai hospital between 2009 to 2015.** CAE = coronary artery ectasia; CAG = coronary artery angiography.


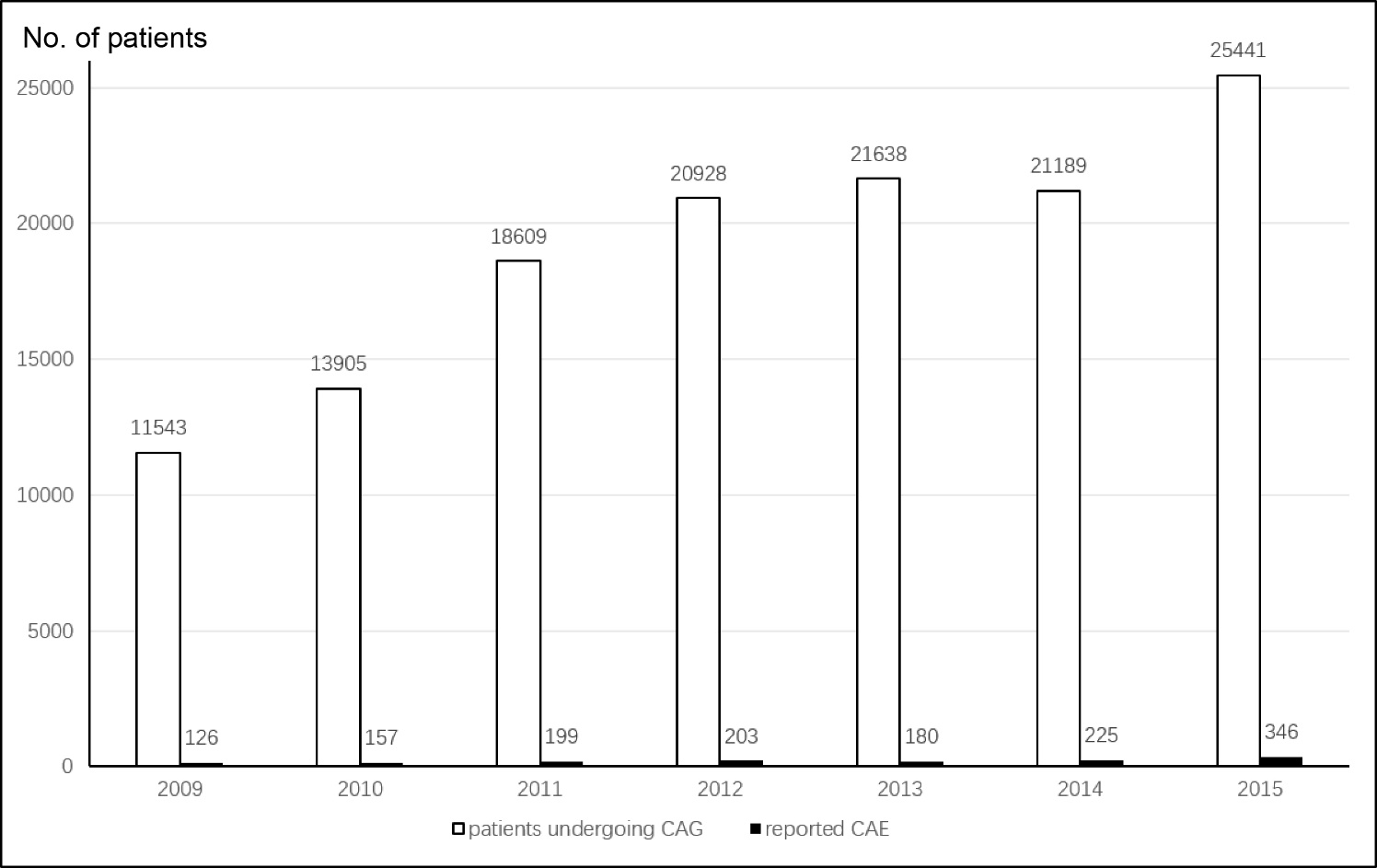


**Supplementary Figure 2. Receiver operating characteristic (ROC) curve of plasma big ET-1 (A) and diffuse dilation (B) and a combination of diffuse dilation and big ET-1 (C) for predicting 5-year MACE.**


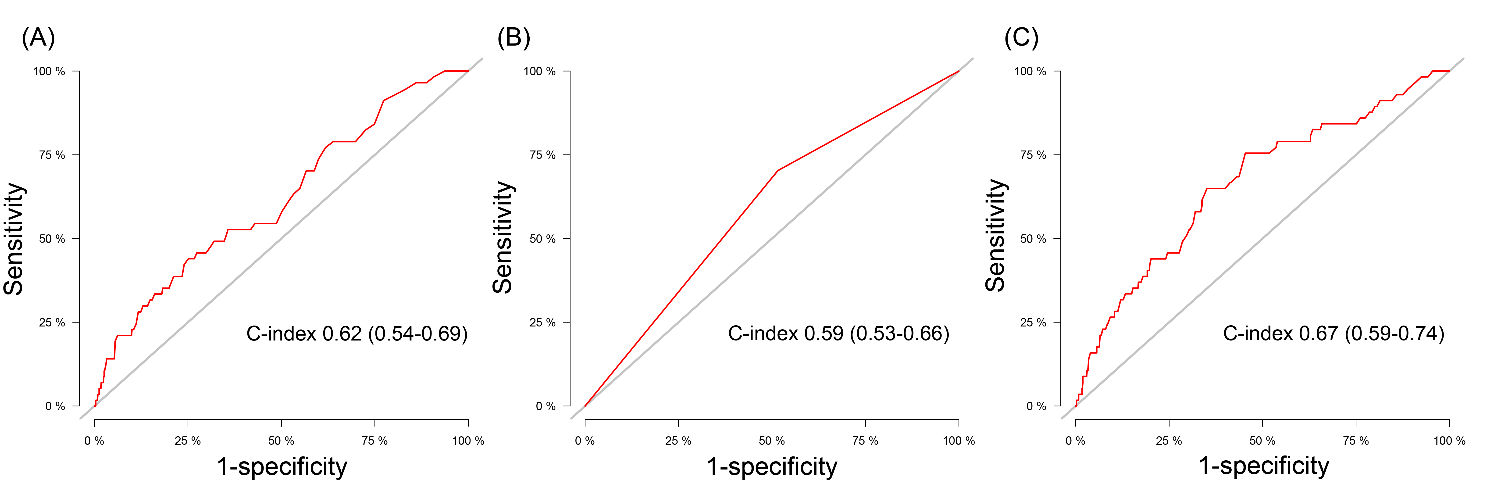


**Supplementary Figure 3.** **the potential effects of ET-1 on vascular endothelial cells, vascular smooth muscle cells and fibroblasts in the pathogenesis of vascular disease.** ET: endothelin. MMP: matrix metalloproteinase.


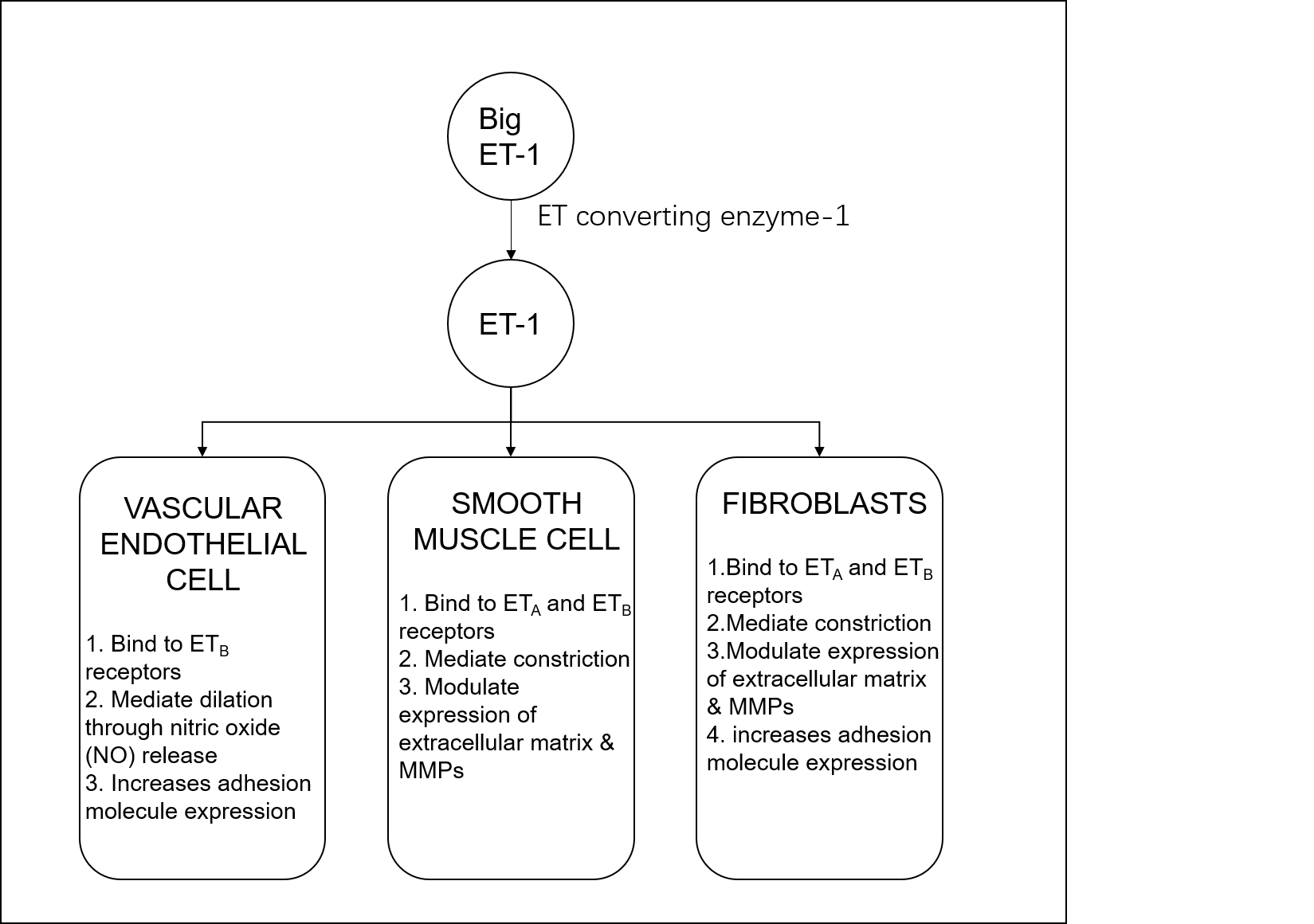


## Supplementary Tables

**Supplementary Table 1. Coronary artery reference diameter in 231 age-sex-matched angiographically normal subjects.**

| Coronary artery segment | reference diameter (mm) |
| --- | --- |
| Right coronary artery |  |
| Proximal | 3.7±0.6 |
| Middle | 3.1±0.5 |
| Distal | 2.8±0.5 |
| Posterior descending | 1.7±0.4 |
| Posterolateral branch | 1.9±0.4 |
| Left anterior descending artery |  |
| Proximal | 3.2±0.6 |
| Middle | 2.8±0.5 |
| Distal | 2.0±0.4 |
| Left circumflex artery |  |
| Proximal | 3.1±0.6 |
| Distal | 2.4±0.4 |
| Obtuse marginal | 1.9±0.5 |
| Left main | 4.4±0.6 |

Values are mean ± SD.
